# Microbial Regulation of p53 Tumor Suppressor

**DOI:** 10.1371/journal.ppat.1005099

**Published:** 2015-09-17

**Authors:** Alexander I. Zaika, Jinxiong Wei, Jennifer M. Noto, Richard M. Peek

**Affiliations:** 1 Department of Veterans Affairs, Tennessee Valley Healthcare System, Nashville, Tennessee, United States of America; 2 Department of Surgery, Vanderbilt University Medical Center, Nashville, Tennessee, United States of America; 3 Department of Cancer Biology, Vanderbilt University Medical Center, Nashville, Tennessee, United States of America; 4 Department of Medicine, Division of Gastroenterology, Vanderbilt University Medical Center, Tennessee, United States of America; Stony Brook University, UNITED STATES

## Abstract

p53 tumor suppressor has been identified as a protein interacting with the large T antigen produced by simian vacuolating virus 40 (SV40). Subsequent research on p53 inhibition by SV40 and other tumor viruses has not only helped to gain a better understanding of viral biology, but also shaped our knowledge of human tumorigenesis. Recent studies have found, however, that inhibition of p53 is not strictly in the realm of viruses. Some bacterial pathogens also actively inhibit p53 protein and induce its degradation, resulting in alteration of cellular stress responses. This phenomenon was initially characterized in gastric epithelial cells infected with *Helicobacter pylori*, a bacterial pathogen that commonly infects the human stomach and is strongly linked to gastric cancer. Besides *H*. *pylori*, a number of other bacterial species were recently discovered to inhibit p53. These findings provide novel insights into host–bacteria interactions and tumorigenesis associated with bacterial infections.

## Author Summary

This review focuses on a novel aspect of host–bacteria interactions: the direct interplay between bacterial pathogens and tumor suppression mechanisms that protect the host from cancer development. Recent studies revealed that various pathogenic bacteria actively inhibit the major tumor suppression pathway mediated by p53 protein that plays a key role in the regulation of multiple cellular stress responses and prevention of cancerogenesis. Bacterial degradation of p53 was first discovered in the context of *Helicobacter pylori* infection, which is currently the strongest known risk factor for adenocarcinoma of the stomach. This phenomenon, however, is not limited to *H*. *pylori*, and many other bacterial pathogens inhibit p53 using various mechanisms. Inhibition of p53 by bacteria is linked to bacterial modulation of the host cellular responses to DNA damage, metabolic stress, and, potentially, other stressors. This is a dynamic area of research that will continue to evolve and make important contributions to a better understanding of host–microbe interactions and tumorigenesis. These studies may offer new molecular targets and opportunities for drug development.

## Historical Perspective of Microbial Inhibition of p53

**Fig 1:**
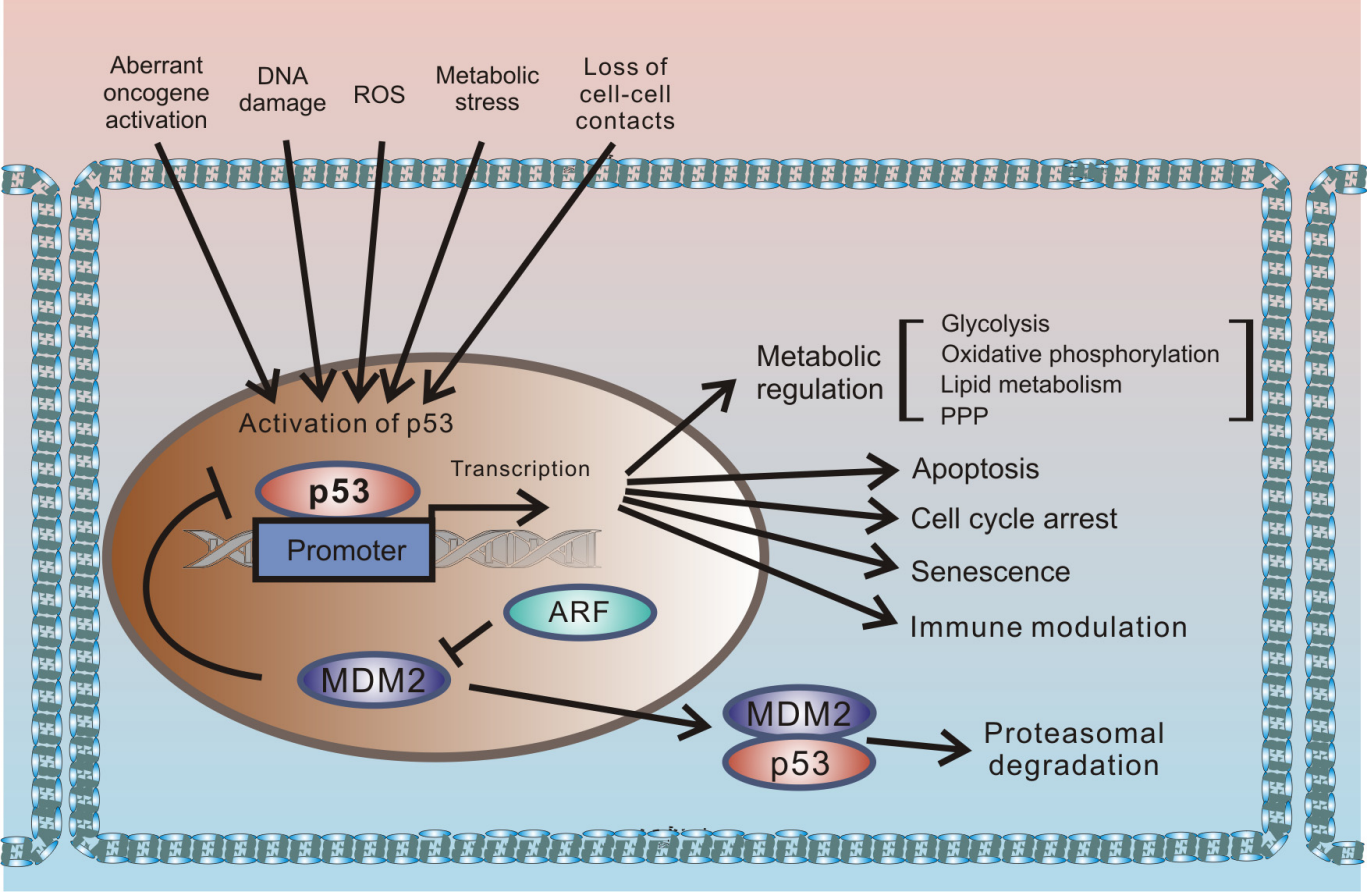
Outline of the regulation of cellular stresses by p53. p53 protein is induced by multiple cellular stresses leading to transcriptional up-regulation of p53 target genes that are involved in regulation of apoptosis, proliferation, metabolism, and immune response. Under normal (unstressed) conditions, levels of p53 protein are tightly controlled by HDM2 E3 ubiquitin ligase, which ubiquitinates p53 leading to its proteasomal degradation. The p14ARF tumor suppressor, which functions upstream of HDM2 and p53, is required for accumulation of p53 under oncogenic stress. The role of p14ARF is to inhibit proteasomal degradation of p53 by sequestering the HDM2 protein in the nucleoli and inhibiting its E3 ligase activity.

p53 protein has been receiving significant attention for more than 30 years. This interest originates from the protein’s prominent role in tumor suppression that was eloquently paraphrased in the scientific literature as “the guardian of the genome” [1]. p53 is a key component of the cellular mechanisms controlling cellular responses to various cellular stresses, including DNA damage, aberrant oncogene activation, loss of normal cell–cell contacts, nutrient deprivation, and abnormal reactive oxygen species (ROS) production. Following cellular stresses, p53 is activated and primarily functions as a transcriptional regulator of expression of multiple effector proteins and miRNAs, which, in turn, regulate key cellular processes such as apoptosis, cellular proliferation, and autophagy. Since regulation of cellular stress responses is tightly intertwined with metabolic regulation, there is an interplay between p53 and multiple pathways involved in regulation of metabolism and cellular homeostasis that is complex and not fully understood. One prominent example is a reciprocal signaling between p53 and mTOR [2]. The latter pathway plays a key role in cell growth and proliferation. p53 is also directly involved in regulation of the cellular energy metabolism and the redox balance regulating glycolysis, oxidative phosphorylation, and the pentose phosphate pathway (PPP). Through multiple mechanisms, p53 can dampen glycolysis and the PPP and promote oxidative phosphorylation. The metabolic functions of p53 are likely to significantly contribute to its tumor suppression activity (Fig 1).

Inactivation of p53 is a hallmark of tumorigenic changes. More than half of all tumors carry p53 mutations, rendering the p53 gene (*tp53*) the most mutated gene in human tumors. p53 can also be inhibited by mutation-independent mechanisms. Inhibition of wild-type p53 by the SV40 virus was one of the first reported examples. SV40 is a small DNA tumor polyomavirus that induces cellular transformation in cell culture and an array of different tumors in animals. In infected cells, viral protein (SV40 large T antigen [T-Ag]) binds p53 and inhibits p53-dependent transcription, resulting in accumulation of inactivated p53 protein [3,4]. Inhibition of p53 by large T-Ag is closely linked to the ability of the SV40 virus to induce tumorigenic transformation; SV40 mutants, which are defective in inhibition of p53, are also defective in cellular immortalization and transformation [5,6].

p53 by itself was originally identified as a protein binding to SV40 large T-Ag [7,8]. Later studies have shown that SV40 T-Ag is not unique in this sense, and other small tumor DNA viruses (adenoviruses and papillomaviruses) also produce similar proteins (E1B-55K and E6) that interact with p53 [9,10]. Although adenoviral protein E1B-55K and human papillomavirus (HPV) protein E6 are different in their amino acid sequences, they converge at the same function, forming protein complexes with p53 to inhibit its activity. HPV and adenovirus (Ad) can also induce ubiquitination and proteasomal degradation of p53 [11]. The ability to degrade p53 varies among viruses. For example, high-risk genital HPV types 16 and 18, which cause around 70% of cervical cancers, efficiently degrade p53, while low-risk viruses such as HPV types 6 and 11 are unable to do so [12,13]. Similarly, p53 is degraded by human adenovirus serotypes 12 and 5 (Ad12, Ad5), while Ad9 and Ad11 do not have this ability [14,15]. To degrade p53, both HPV and Ad use the host protein degradation machinery. HPV E6 protein interacts with the host E3 ubiquitin ligase, E6AP, causing its substrate specificity to be altered so that it ubiquitinates p53 and induces its degradation by the 26S proteasomes [16]. In Ad-infected cells, viral proteins E1B-55K and E4orf6 interact with cellular proteins Cullin5 (or Cullin2), Rbx1, and Elongins B and C to form a Cullin-containing E3 ubiquitin ligase that targets p53 for proteasomal degradation [14,17,18]. A similar degradation strategy is also used by the Epstein–Barr virus (EBV), which forms a complex containing viral protein BZLF1 and cellular Cullin2/5-containing E3 ubiquitin ligase to degrade p53 [19].

Due to a relatively simple organization of the viral genomes, viruses have to rely on host resources for most aspects of their life cycle. In the process of interacting with host cells, they alter the intracellular environment to make it suitable for viral replication. These drastic alterations, however, may cause cellular stress and activate p53, resulting in cell cycle arrest or apoptosis of host cells; both outcomes are detrimental to viral replication. It is plausible that inhibiting p53 may provide advantages to viruses that have evolved to do so. Recently, this concept was further expanded to include additional microorganisms. These novel data are discussed in this review, focusing on specific mechanisms of bacterial inhibition of p53.

## If Viruses Can Do It, Why Can’t Other, More Complex Microorganisms?

Recent studies have found that it is not only viruses, but also some pathogenic bacteria, that actively inhibit p53 and induce its degradation. This phenomenon was initially described in gastric cells co-cultured with *Helicobacter pylori* [20]. *H*. *pylori* is a gram-negative, spiral-shaped pathogen that lives in the stomachs of approximately half of the world’s population. The infection is typically acquired during childhood and causes lifelong chronic infection. Because of the association between *H*. *pylori* infection and the incidence of gastric cancer, the International Agency for Research on Cancer (IARC) has classified this bacterium as a Group 1 carcinogen. *H*. *pylori* infection is considered to be the strongest known risk factor for gastric cancer, and epidemiological studies have estimated that, in the absence of *H*. *pylori*, 75% of gastric cancers would not occur [21].

**Fig 2:**
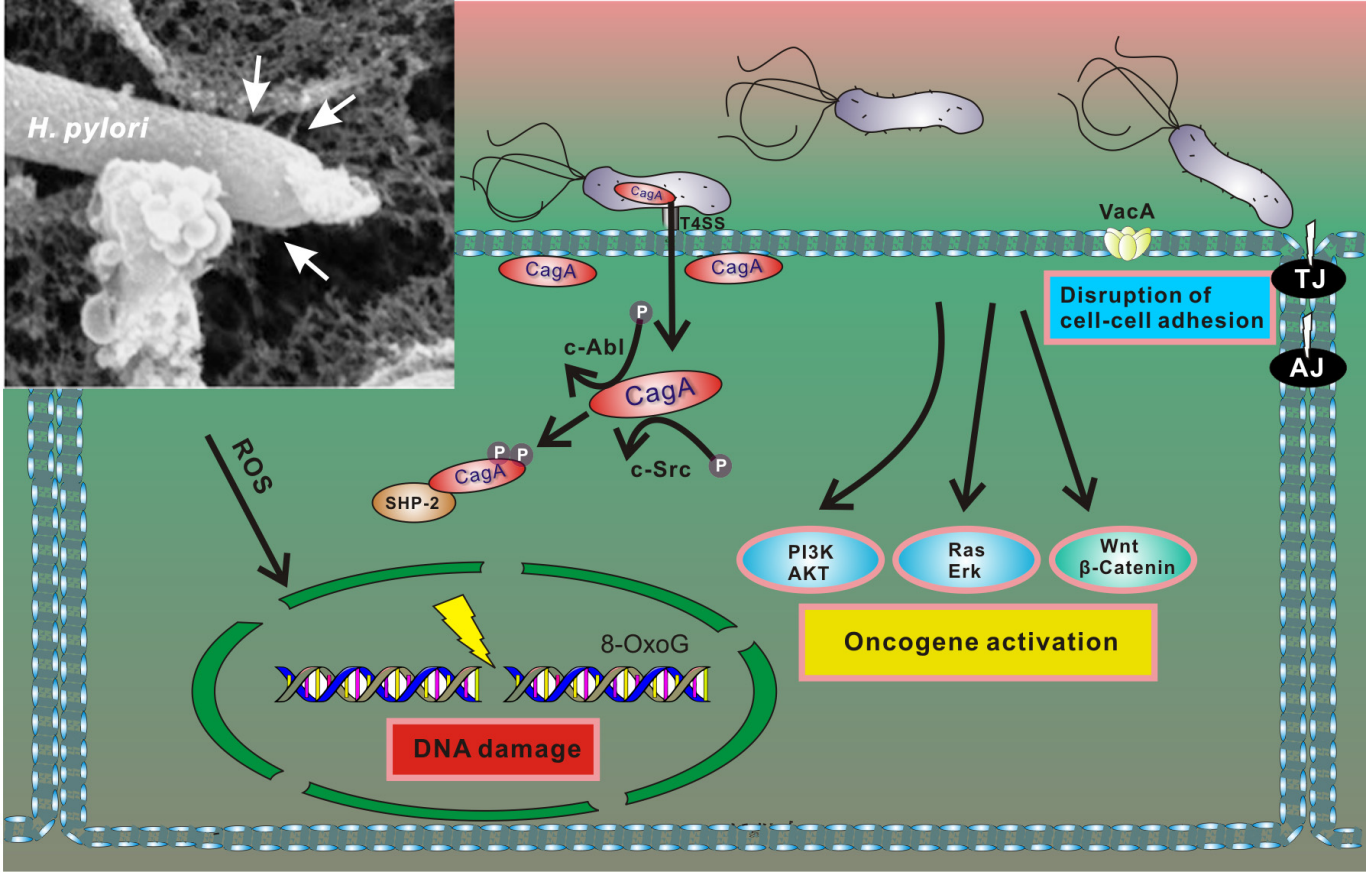
Interaction between *H*. *pylori* and gastric epithelial cells results in cellular stress. After adherence, *H*. *pylori* translocates CagA protein into host cells using the T4SS. Translocated CagA is rapidly tyrosine phosphorylated by host kinases c-Src and c-Abl and binds to SHP2 phosphatase, leading to alteration of intracellular signaling, including activation of multiple oncogenic pathways and cytoskeletal rearrangement [22]. *H*. *pylori* also produces VacA toxin, which binds to the cell surface and forms oligomers. VacA is internalized and forms anion-selective channels in the membranes of endocytic compartments, resulting in cell vacuolation. In addition, *H*. *pylori* compromises the integrity of the host genome by inducing oxidative DNA damage and DNA double-strand breaks [27,28]. Insert: An electron microphotograph of *H*. *pylori* attached to the surface of AGS human gastric epithelial cells. AGS cells were co-cultured with *H*. *pylori* strain 26695, and cag T4SS pili were visualized by scanning electron microscopy (white arrows).

Pathogenesis associated with *H*. *pylori* infection is determined by interactions between bacterial factors and host cells. The most well characterized bacterial virulence determinants are the vacuolating cytotoxin A (*vacA*) and the *cag* pathogenicity island (*cag PAI*). The *cag PAI* is a 40 kb region of DNA that encodes a type IV secretion system (T4SS) that forms a syringe-like pilus structure used for the injection of a bacterial protein CagA (cytotoxin-associated gene A) into gastric cells. Following the delivery, intracellular CagA is localized to the plasma membrane and triggers complex alterations of the host signaling pathways [22], including activation of cellular oncogenes (Fig 2). CagA itself functions as an oncoprotein. In laboratory tests, CagA promoted anchorage-independent growth and, when transgenically expressed in mice, led to spontaneous development of gastrointestinal and hematopoietic neoplasms [23,24]. Oncogenic potential of CagA has also been demonstrated using Drosophila and zebrafish experimental models [25,26].

*H*. *pylori* infection results in conditions of cellular stress because the bacteria induce DNA damage and disturb normal cellular homeostasis (including aberrant activation of multiple oncogenic pathways), all of which are conditions that typically activate p53 [27,28]. However, initial studies of the p53 stress response revealed that *H*. *pylori* is able to dampen activity of p53 protein by inducing its rapid degradation [20]. The ability of *H*. *pylori* to suppress the p53 response was also demonstrated when DNA damage was experimentally induced by DNA-damaging agents [20,29,30]. The bacteria specifically target p53, as p73—another member of the p53 protein family, which has significant functional and structural similarities to p53—is not down-regulated by *H*. *pylori* but rather induced [31]. The ability to induce degradation of p53 varies between *H*. *pylori* strains, with CagA-positive bacteria being more potent [20,29]. Although CagA likely does not directly bind to p53, it induces its degradation [29]. Notably, ectopic transfection of CagA is sufficient to inhibit p53 activity and induce its degradation [20,30]. Recent studies pointed out a complex nature of CagA–p53 interactions. It was shown that levels and natural variability of CagA protein highly affect p53 degradation [32]. Among other bacterial factors, VacA was also reported to regulate p53 [33–35]. Down-regulation of p53 was found to facilitate autophagy in infected cells [35].

The kinetics of p53 in infected cells in vivo appears to be complex. In infected Mongolian gerbils, which are commonly used for studies of *H*. *pylori* infection, expression of p53 was changed in a bimodal fashion, with an accumulation after initial infection that was followed by a rapid down-regulation of p53 protein in gastric epithelial cells. A second peak of p53 was observed later, when gastritis (inflammation of the lining of the stomach) developed. These findings led to a hypothesis that, at a certain time, levels of p53 reflect a balance between p53 degradation induced by the bacteria and p53 induction caused by cellular stress [20]. A down-regulation of p53 protein, but not p53 mRNA, was observed in *H*. *pylori*-infected mice [36].

In contrast to small DNA tumor viruses, *H*. *pylori* takes advantage of host mechanisms normally regulating p53 [20,35]. The bacteria enhance proteasomal degradation of p53 mediated by E3 ubiquitin ligase HDM2 by increasing its phosphorylation at serine 166. An increased phosphorylation of HDM2 was found in gastric epithelial cells co-cultured with *H*. *pylori* in vitro and *H*. *pylori*-infected animals and humans in vivo [20,35,37]. Inhibition of HDM2 activity with siRNA or chemical inhibitor Nutlin3 suppresses bacterial degradation of p53 [20,35,38]. A similar effect can be achieved by inhibition of Akt and Erk kinases, showing that these enzymes mediate phosphorylation of HDM2 protein in infected cells [35,38]. Expression of HDM2 was found to correlate with phosphorylated Akt (pAkt) in patients infected with *H*. *pylori* [37]. In addition to HDM2, recent studies reported that another cellular E3 ubiquitin ligase, Mule/ARF-BP1, is involved in degradation of p53 in *H*. *pylori*-infected cells [32]. It remains unclear how this enzyme is activated by the bacteria.

**Fig 3:**
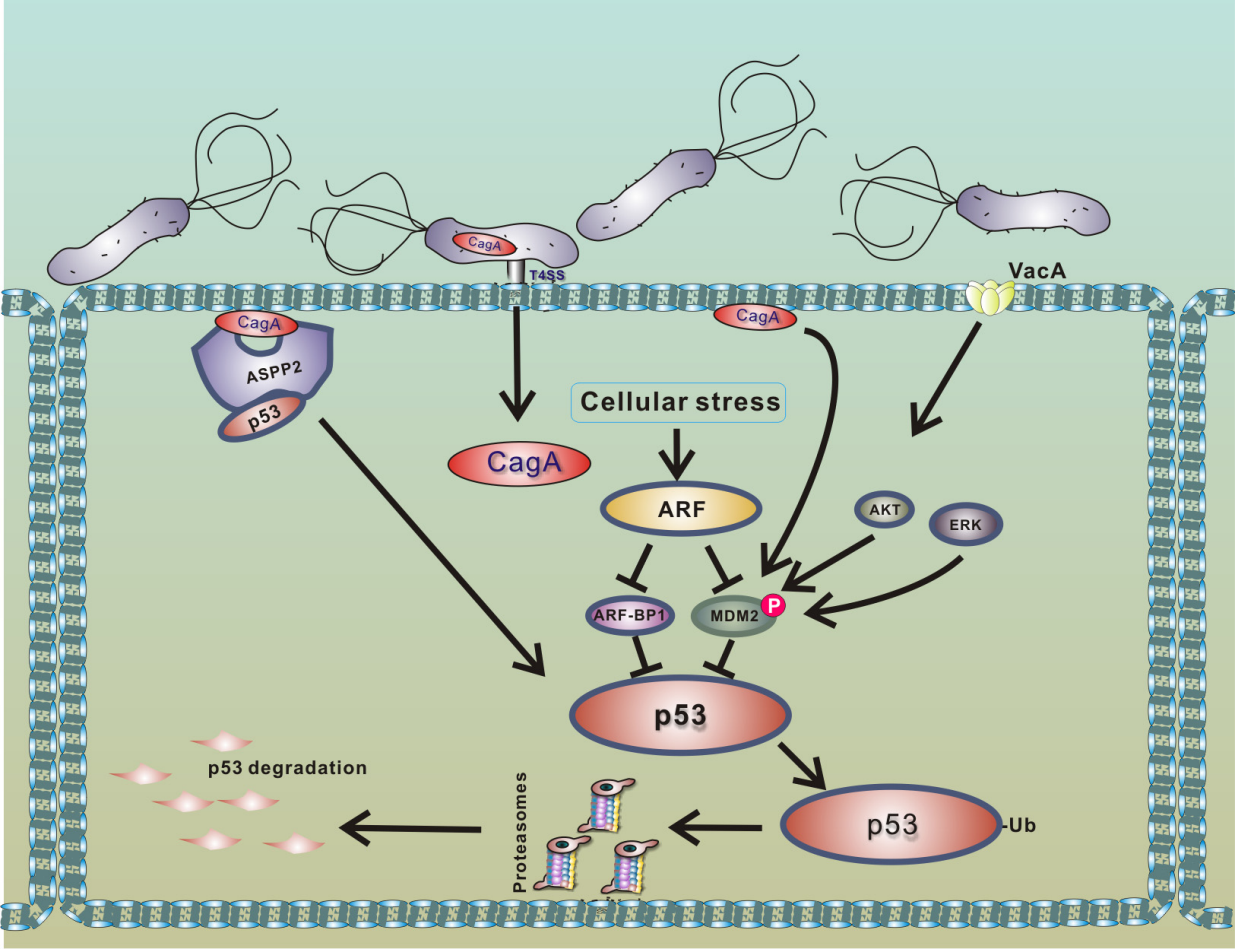
Model of p53 down-regulation by *H*. *pylori*. Interaction of *H*. *pylori* with gastric epithelial cells leads to translocation of bacterial CagA protein into host cells and activation of PKB/Akt and Erk kinases, which phosphorylate HDM2 protein [20,35,38]. The consequent activation of HDM2 and ARF-BP1 E3 protein ligases induces a rapid degradation of p53 protein. Binding of CagA to ASPP2 protein facilitates this process [29]. Degradation of p53 is strongly suppressed in cells expressing functional p14ARF, since ARF inhibits activities of MDM2 and ARF-BP1 proteins [32].

p14ARF tumor suppressor (termed p19ARF in rodents and p14ARF in humans), which functions upstream of p53, was found to be a critical modulator of p53 protein stability in infected cells [32], as ARF inhibits activities of both HDM2 and ARF-BP1 proteins [39–41]. It was shown that cells expressing functional ARF are significantly more resistant to degradation of p53 (Fig 3). However, when ARF protein levels are decreased due to hypermethylation or deletion of the ink4a/ARF locus, *H*. *pylori* efficiently degrades p53 [32]. Loss of ARF occurs during gastric tumorigenesis and can be found in gastric precancerous lesions. Methylation of the p14ARF gene is also increased with age [42]. Given these findings, it was hypothesized that older people with gastric precancerous lesions, who are infected with *H*. *pylori*, may be particularly vulnerable to degradation of p53 [32].

Among other cellular factors, ASPP2 protein (apoptosis-stimulating protein of p53), which normally activates p53, was identified to regulate p53 in *H*. *pylori*-infected cells [29]. Buti et al. showed that binding of CagA protein to ASPP2 results in inhibition of transcriptional and proapoptotic activities of p53 and induction of proteasomal degradation of p53.

Recent studies suggest that bacterial degradation of p53 may contribute to gastric tumorigenesis. It was reported that clinical isolates of *H*. *pylori* varied greatly in their ability to degrade p53, but that, generally, isolates associated with a higher gastric cancer risk more strongly affect p53 when compared to low-risk counterparts [32].

*H*. *pylori* inhibits p53 through multiple mechanisms, implying that inhibition of p53 activity is an important factor for successful infection. The bacteria not only induce degradation of p53, but also alter the expression profile of p53 isoforms [43]. Interaction of *H*. *pylori* with gastric epithelial cells, mediated via the cag PAI, induces N-terminally truncated Δ133p53 and Δ160p53 isoforms, which inhibit transcriptional and proapoptotic activities of p53, resulting in activation of NFkB. Induction of proinflammatory cytokine Macrophage Migration Inhibitory Factor (MIF) by *H*. *pylori* was suggested to inhibit p53 by decreasing its phosphorylation [44]. It was also shown that *H*. *pylori* can facilitate mutagenesis of the *p53* gene. Infection with *H*. *pylori* leads to aberrant induction of activation-induced cytidine deaminase (AID), which deaminates cytosine residues, leading to accumulation of p53 mutations in gastric tissues [45]. Interestingly, AID and other cytidine deaminases are induced by a number of viruses such as HPV, HTLV-1, HCV, and others [46–48]. SV40 and influenza A viruses have been shown to affect expression of p53 isoforms [49,50].

**Fig 4:**
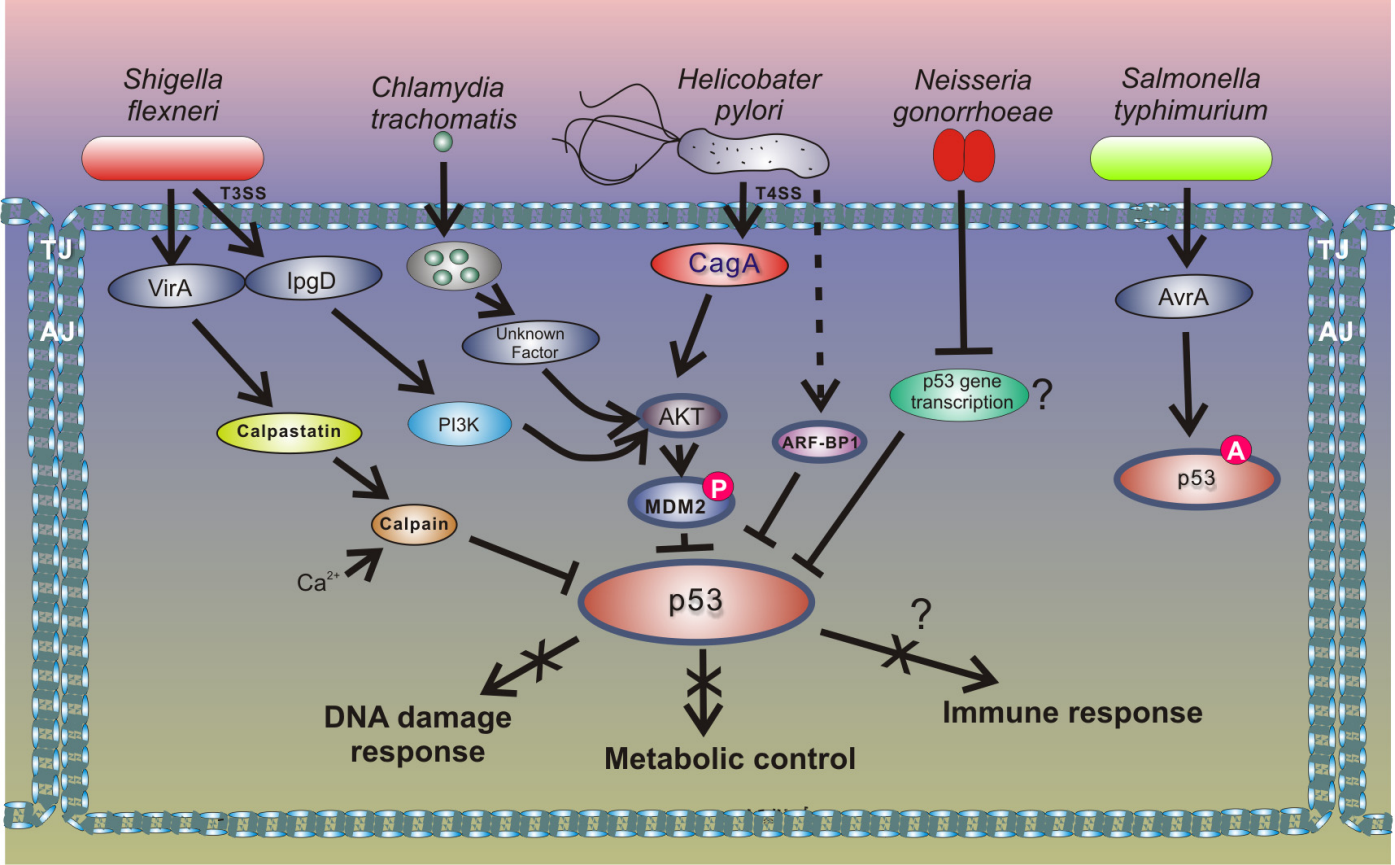
Outline of the interactions between bacterial pathogens and the p53 pathway.

A new and exciting development in this area is that other bacteria induce degradation of p53 using a similar mechanism to that of *H*. *pylori* (Fig 4). Two research groups have recently reported that the intracellular bacterial pathogen *Chlamydia trachomatis*, and potentially other *Chlamydia* species, induces degradation of p53 by activating HDM2 protein [51,52]. *C*. *trachomatis* is a common cause of bacterial sexually transmitted disease (STD) and blinding trachoma. Similar to *H*. *pylori*, *C*. *trachomatis* activates the PI3K/Akt pathway and increases phosphorylation of HDM2 (Ser166), leading to activation of HDM2 and proteasomal degradation of p53. Down-regulation of p53 allows *Chlamydia* to enhance activity of the PPP that provides bacteria with necessary metabolites, such as nucleotides precursors, and protects against oxidative stress by increasing the cellular NADPH pool [52]. Enforced expression of p53 in infected cells results in strong inhibition of chlamydial growth, while overexpression of glucose-6-P-dehydrogenase, a key enzyme in the PPP that is inhibited by p53, rescues the bacterial growth. The authors reported that degradation of p53 by *Chlamydia* interferes with the host’s response to genotoxic stress and may contribute to cancerogenesis in the female genital tract [51,52].

Inhibition of p53 through the HDM2-dependent mechanism is also employed by enteropathogen *Shigella flexneri*, which causes bacillary dysentery in humans. Infection with *Shigella* is accompanied by strong genotoxic stress and cellular damage [53]. To prevent activation of p53, *Shigella* causes rapid degradation of p53 using two distinct mechanisms. During the early phase of infection, the bacterial virulence effector IpgD promotes activation of the host PI3K/Akt pathway and phosphorylation of HDM2 at serines 166 and 186, causing activation of HDM2 and degradation of p53. The second mechanism for p53 inhibition comes into play during the late phase of infection. p53 is proteolytically cleaved by the calpain protease system, in which activation is facilitated by the *Shigella* virulence effector VirA. The VirA activates calpain by promoting proteolysis of the calpain inhibitor calpastatin. Bergounioux et al. suggested that *Shigella* inhibits p53 to prevent apoptotic cells death that saves energy and preserves its own epithelial niche [53]. Interestingly, not all enteric pathogens inhibit p53. Activation of p53 was reported in the context of *Salmonella typhimurium* infection [54]. Outside the *Enterobacteriaceae* family, down-regulation of p53 protein was reported in studies of *Neisseria gonorrhoeae*, which is responsible for the sexually transmitted gonorrhea that may increase the risk of genital neoplasms [55]. Similar to the aforementioned pathogens, *N*. *gonorrhoeae* causes strong genotoxic stress and induces both single and double strand DNA breaks. The mechanism of p53 down-regulation is not fully understood, but Vielfort et al. reported that the bacteria can inhibit transcription of the p53 gene [56].

Inhibition of p53 may provide certain benefits to bacteria. One particular mechanism that may be targeted by bacteria is the p53 DNA damage response. Inhibition of p53 may allow bacteria to subvert the host cell cycle control and apoptosis mechanisms, resulting in inhibition of cell death and survival of host cells damaged by infection. This is in agreement with the findings of antiapoptotic and prosurvival effects produced by bacterial pathogens, which inhibit p53 [20,29,52,53]. In the case of *H*. *pylori*, expression of the CagA virulence factor is sufficient to inhibit p53 and extend short and long term survival of gastric epithelial cells that underwent DNA damage [20]. Besides the DNA damage response, bacteria may also target the metabolic control of p53. Inhibition of the p53 metabolic regulation may be particularly important for obligatory intracellular pathogens such as *Chlamydia*. As described above, degradation of p53 allows *C*. *trachomatis* to release inhibition of the PPP elicited by p53. When bacterial degradation of p53 was experimentally inhibited, the development and formation of infectious progeny was blocked, suggesting that metabolic control of p53 provides antibacterial protection. It is possible to draw a parallel between *Chlamydiae* and viruses since both are obligatory intracellular pathogens, which strictly rely on the host resources. Similar to viruses, inhibition of p53 allows *Chlamydia* to reprogram the host cell signaling to create a metabolic environment necessary for chlamydial survival and growth. To some extent, this may also be applied to obligate parasitic *Mycoplasma* bacteria, which inhibit activity of p53 [57]. A more complex picture emerges in regards to the role of the p53 signaling in the context of chronic infections with extracellular pathogens such as *H*. *pylori*. One proposed possibility is that inhibition of p53 helps *H*. *pylori* to compromise the gastric epithelial barrier, allowing the bacteria to acquire nutrients from the host or get access to the lamina propria. This concept is supported by recent findings showing that *H*. *pylori* inhibits activation of p53 induced by disruption of the adherens junctions, which stabilize cell–cell adhesion [38]. It was also suggested that suppression of p53 responses may help *H*. *pylori* adapt during the early phase of infection and prevent the host immune response [20]. The p53 pathway is known to affect immune response [58]. Among direct transcription targets of p53 are a number of proteins regulating innate immunity and cytokine and chemokine production. p53 is also known to affect NF-κB activity and pro-inflammatory signaling. Although immunomodulatory function may play a role, there is no direct evidence yet that bacterial inhibition of p53 affects the host immune response. Additional studies are needed to further explore these mechanisms.

## Summary

Interaction of bacterial pathogens with the host cells induces DNA damage, alters intracellular signaling, and profoundly affects normal cellular homeostasis. To prevent the cellular stress response, which may be detrimental to a successful infection, some bacteria have evolved to inhibit p53, a key component of the stress response machinery. Bacteria inhibit p53 through multiple mechanisms, including protein degradation, transcriptional inhibition, and post-translational modifications. Current research revealed that p53 has a role in controlling the bacterial infections and that inhibition of p53 may confer certain selective advantages to bacteria. Unfortunately, this may have grave consequences for the hosts, increasing the risk of tumor development. It is particularly relevant to prolonged chronic infections. Initial experiments with inhibition of protein degradation of p53 demonstrate that p53 activities can be restored in infected cells using specific chemical inhibitors. These findings may offer new and exciting opportunities for therapeutic targeting of p53 in infected cells. Future studie*s* of the bacterial regulation of p53 hold the promise of a better understanding of pathogenesis and tumorigenesis associated with bacterial infections.
